# The Function of *LmPrx6* in Diapause Regulation in *Locusta migratoria* Through the Insulin Signaling Pathway

**DOI:** 10.3390/insects11110763

**Published:** 2020-11-05

**Authors:** Jun Chen, Dong-Nan Cui, Hidayat Ullah, Shuang Li, Fan Pan, Chao-Min Xu, Xiong-Bing Tu, Ze-Hua Zhang

**Affiliations:** 1 State Key Laboratory for Biology of Plant Diseases and Insect Pests, Institute of Plant Protection, Chinese Academy of Agricultural Sciences, Beijing 100193, China; cjhp2014@163.com (J.C.); Cuidongnan88@163.com (D.-N.C.); shabkadar@yahoo.com (H.U.); sclishuang61@163.com (S.L.); lillianP@163.com (F.P.); 18363972673@163.com (C.-M.X.); 2 Department of Agriculture, The University of Swabi, Anbar 23561, Pakistan; 3 College of Animal Science and Technology, Hebei Agricultural University, Baoding 071001, China

**Keywords:** LmPrx6, RNA interference, ISP, diapause rate

## Abstract

**Simple Summary:**

LmPrx6 of the insulin signaling pathway is significantly associated with diapause induction in *Locusta migratoria* L. as per our pervious transcriptome data. In the current study, we first cloned and sequenced the gene and demonstrated its similarity to other Prxs using phylogenetic analyses. Later on, we knocked down Prx6 using RNAi and showed that phosphorylation of proteins associated with the insulin signaling pathway and responses to oxidative stress were altered. Knockdown of Prx6 also resulted in a reduced ability to enter diapause, and hence, we are of the opinion that this gene could serve as an effective target for RNAi-based control of *L. migratoria* L. The study has provided some helpful insights into the diversified roles of Prx6 in locusts and will be of interest to other insect pests for examining the relatively unexplored group of proteins as well.

**Abstract:**

Peroxiredoxins (Prxs), which scavenge reactive oxygen species (ROS), are cysteine-dependent peroxide reductases that group into six structurally discernable classes: AhpC-Prx1, BCP-PrxQ, Prx5, Prx6, Tpx, and AhpE. A previous study showed that forkhead box protein O (FOXO) in the insulin signaling pathway (ISP) plays a vital role in regulating locust diapause by phosphorylation, which can be promoted by the high level of ROS. Furthermore, the analysis of transcriptome between diapause and non-diapause phenotypes showed that one of the Prxs, LmPrx6, which belongs to the Prx6 class, was involved. We presumed that *LmPrx6* might play a critical role in diapause induction of *Locusta migratoria* and LmPrx6 may therefore provide a useful target of control methods based on RNA interference (RNAi). To verify our hypothesis, *LmPrx6* was initially cloned from *L. migratoria* to make *dsLmPrx6* and four important targets were tested, including protein-tyrosine phosphorylase 1B (LmPTP1B), insulin receptor (LmIR), RAC serine/threonine-protein kinase (LmAKT), and LmFOXO in ISP. When *LmPrx6* was knocked down, the diapause rate was significantly reduced. The phosphorylation level of LmPTP1B significantly decreased while the phosphorylation levels of LmIR, LmAKT, and LmFOXO were significantly increased. Moreover, we identified the effect on two categories of genes downstream of LmFOXO, including stress tolerance and storage of energy reserves. Results showed that the mRNA levels of catalase and Mn superoxide dismutase (Mn-SOD), which enhanced stress tolerance, were significantly downregulated after silencing of *LmPrx6*. The mRNA levels of glycogen synthase and phosphoenolpyruvate carboxy kinase (PEPCK) that influence energy storage were also downregulated after knocking down of *LmPrx6*. The silencing of LmPrx6 indicates that this regulatory protein may probably be an ideal target for RNAi-based diapause control of *L. migratoria*.

## 1. Introduction

Insects have evolved diapause to adapt to seasonally unfavorable environments [1]. Diapause not only enables insects to escape a harsh natural environment but also allows the insect population to develop to a consistent stage, such as the same instar, thereby increasing the possibility of male–female pairing, ensuring highly efficient reproduction [2]. Moreover, diapause, the process opposite to those of reproductive growth, including arrest or slowing of cell division in response to anticipated stress, thereby reducing metabolism and enhancing stress tolerance [3]. *Locusta migratoria* is one of the most important agricultural pests worldwide and in autumn adults (Huanghua strain) in Huanghua, Tianjin, China (38°49′N, 117°18′E) prefer to diapause during overwintering, though diapause varies in different geographic locations [1]. The diapause induction in *L. migratoria* is a trans-generational process from maternal parents to their offspring induced by short day (light:dark = 10:14) as a maternal effect, which makes the *L. migratoria* as one of the most important model insects for investigating the mechanism of insect diapause induction [4,5,6].

**Figure 1:**
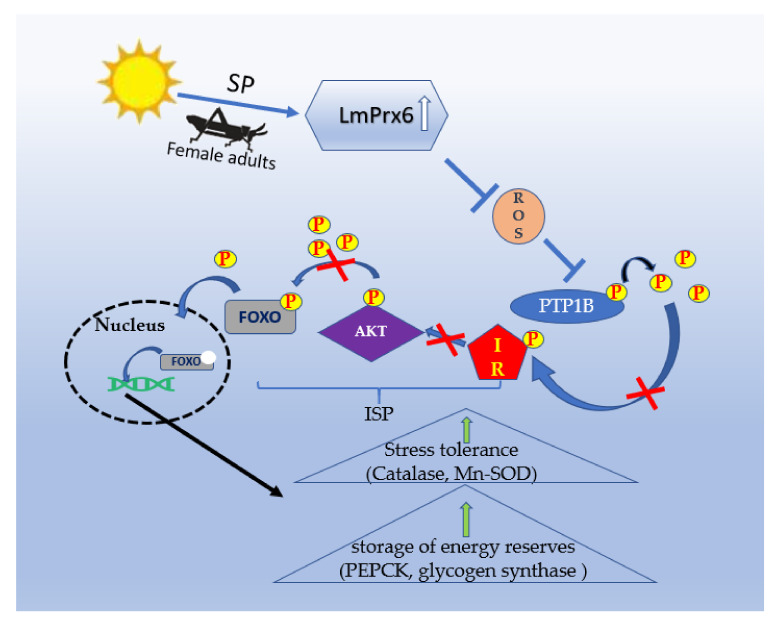
The hypothetical molecular relationships of LmPrx6 (Prx6 of *Locust migratoria*) in ISP in an adult female: Female adults sense the short photoperiod (SP) and cause enhanced LmPrx6 that inhibits ROS. The reduction of ROS leads to the phosphorylation of PTP1B. The phosphorylated LmPTP1B inhibits IR, which could phosphorylate AKT. The attenuation of AKT leads to the dephosphorylation of FOXO. Then, the dephosphorylated FOXO transfers to the nucleus to induce the expression of genes of stress tolerance (catalase and Mn-SOD) and genes of storage of energy reserves (phosphoenolpyruvate carboxy kinase (PEPCK) and glycogen synthase).

Our previous study showed that FOXO in ISP plays a vital role in regulating locust diapause by phosphorylation [7], which can be promoted by a high level of ROS [8]. In *C. elegans* [9] and *D. melanogaster* [10], genetic screens identified FOXO as a key signaling pathway regulating lifespan. Studies on the target of insulin signaling, daf-16/FOXO, suggest that dauer arrest and lifespan are regulated by FOXO activation [11]. The *C. elegans* FOXO is a critical target of the insulin/IGF-1 signaling pathway that mediates stress resistance [12]. After knocking down FOXO transcript by injection of dsRNA into these diapausing mosquitoes, there is an immediate halt in the accumulation of lipid reserves [13]. A previous study showed that the presence of ROS regulates the insulin pathway during fat synthesis [14]. A large amount of ROS can activate NADPH oxidase 4 (Nox4) in early insulin-induced fat synthesis and inhibit the activity of protein-tyrosine phosphorylase 1B (PTP1B) [14,15], which plays an essential role in balancing insulin receptor (IR) and insulin receptor substrate (IRS) [16,17]. Furthermore, IRS can activate proteins downstream of the insulin signaling pathway (ISP), such as RAC serine/threonine-protein kinase (AKT) and forkhead box protein O (FOXO). The activated AKT phosphorylates FOXO downstream of the ISP [18] to mediate diapause, as shown in mosquitoes [19] and silkworms [20]. Therefore, we speculate that there is a similar molecular mechanism of diapause induction in maternal *L. migratoria* when they are influenced by short photoperiod condition (Figure 1). However, the major upstream genes that regulate ROS and FOXO are still unclear and it is more important to investigate these genes.

*L. migratoria* has facultative egg diapause, and its diapause occurs from sensing a short photoperiod at the adult stage to anatrepsis at the egg stage. In this study, we focus on the diapause induction stage or females under a short photoperiod. In the diapause induction stage, the female adults experience the short photoperiod to form diapause signals, which would be transferred to eggs in the ovary to control egg diapause [21]. Prior to the current experiment, analysis of transcriptome differences of the diapause and non-diapause females were carried out [22], and the FPKM of *LmPrx6* in diapause females was 34.9124 versus 9.9304 in non-diapause females. Log_2_FC is about 1.8. It means that *LmPrx6* in diapause females is significantly higher than those of the non-diapause phenotype. However, the mechanism controlling diapause was not clear [22].

Prx6, a 1-Cys-type peroxide reductase with only one active Cys residue in its peptide chain, is oxidized to form Cys-SOH, which is produced as an electron donor, allowing the reaction to move forward and reducing the proportion of ROS [23].

Peroxiredoxins (Prxs), scavengers of reactive oxygen species (ROS) produced by active metabolism [24], are a newly discovered class of antioxidant enzymes in addition to the enzymes superoxide dismutase (SOD) and glutathione peroxidase (GPX). Prxs have an active cysteine residue at the amino terminus, which can function as an electron donor to reduce oxides [25]. Despite limited functional differences, Prxs are still classified into three types by their structures, referred to as typical 2-Cys (Prx I-IV), atypical 2-Cys (Prx V), and 1-Cys (Prx VI) [26]. Using the Deacon Active Site Profiler (DASP) tool, Prxs is classified into six groups: AhpC-Prx1, BCP-PrxQ, Prx5, Prx6, Tpx, and AhpE [27]. The AhpC-Prx1 subfamily is essentially synonymous with the “typical 2-Cys Prxs” and has also been referred to as the “A” group in the plant field [28]. Members of the AhpC-Prx1 subfamily have been linked to important roles in cellular signaling [29] and some appear to be regulated by phosphorylation [30]. The Prx6 subfamily takes its name from the first Prx to be crystallized, human PrxVI [31], formerly referred to as “ORF6”. Prx6 proteins are most similar to the AhpC/Prx1 subfamily, containing a C-terminal extension and forming B-type dimers and, in some cases, higher oligomeric states and the members are predominantly 1-Cys, though 2-Cys representatives exist [26], and the direct reductant of Prx6 subfamily members is generally not known [32]. At present, studies on Prx6 in insects are limited, with studies mainly in humans [33], mice [34], nematodes [35], crustaceans [36], and fish [37].

Broad-spectrum chemical pesticides contaminate a huge amount of farmlands and cause resistant pests, which is not sustainable. A novel environmentally friendly approach, RNAi, could therefore be a desirable alternative to pesticides. To study the role of *LmPrx6*, RNAi, a powerful approach to functional analysis of genes in insects, was applied to analyze the related genes and proteins.

## 2. Materials and Methods

### 2.1. Insect Rearing

The *Locusta migratoria* L. was previously obtained from fields in Huanghua, Tianjin, China (38°49′ N, 117°18′ E) in November 2007. All insects were maintained until the present study in our lab at the State Key Laboratory for Biology of Plant Diseases and Insect Pests, Institute of Plant Protection, Chinese Academy of Agricultural Sciences. New first instars were kept in 40 cm × 40 cm × 40 cm rearing cages and transferred to 20 cm × 20 cm × 28 cm mesh cages until the fourth instar. Then, the cages were placed in artificial climate chambers (PRX-250B-30, Haishu Saifu Experimental Instrument Factory, Ningbo, China). The conditions were at either 27 °C and 60% RH under a long photoperiod of 16:8 L:D to produce non-diapause eggs, and at 27 °C, 60% RH under a short photoperiod of 10:14 L:D to produce diapause eggs.

### 2.2. cDNA Synthesis and LmPrx6 Cloning

The whole body of adult locusts was used to extract total RNA. TRIcom Reagent (Tianmo Biotech, Beijing, China) was used to extract RNA, and cDNA was synthesized according to the PrimeScript^TM^ 1st strand cDNA Synthesis Kit (TaKaRa, Dalian, China). By analyzing the transcriptome of migratory locust, we obtained the sequence of *LmPrx6* (GenBank accession: MT563098) and primers were subsequently designed in DNAMAN6. Using the cDNA of *L. migratoria* as a template, *LmPrx6* was amplified by the specific primers *LmPrx6*-1F and *LmPrx6*-1R (Table 1). The PCR product was purified using a TIANgel Midi Purification Kit (TIANGEN Biotech, Beijing, China) connected to the pMD19-T vector (TaKaRa, Japan). Then, the recombinant was transformed into the Trans1-T1 strain of *Escherichia coli*. A total of 500 μL of LB liquid medium were added to the transformed *E. coli*. Then, the mixture was shaken at 200 rpm and 37 °C for 2 h. Bacterial solution (100 μL) was applied to LB solid medium, which included 1% ampicillin. The medium was incubated at 37 °C for 12 h. Three replicates of the single recombinant colony were transferred into 5 mL of liquid LB culture medium with 1% ampicillin, and shaken for 3–6 h at 37 °C, then the medium was used as a PCR template. Reconstructed plasmid was extracted from the transformed strains. Primers in this experiment were synthesized and reconstructed and the plasmid was sequenced by Sangon Biotech Company Ltd. (Table 1).

### 2.3. Structure and Phylogenetic Analyses of LmPrx6

For subsequent sequence analysis, the Self-Optimized Prediction Method with Alignment (SOPMA) online server (https://npsa-prabi.ibcp.fr/cgi-bin/npsa_automat.pl?page=/NPSA/npsa_sopma.html) was applied to predict the secondary structure of LmPrx6 protein and the tertiary structure was predicted through the website: https://swissmodel.expasy.org/interactive. Signal peptides were predicted as per the methodology of the hidden Markov model of SignalP4.1 (http://www.cbs.dtu.dk/services/SignalP/). Multiple sequence alignment with 15 sequences (NCBI ID were: AEI52300.1, AAX18657.1, XP_001607910.1, AAG47823.1, AAK97814.1, XP_968419.1, ABB91779.1, ACJ53746.1, NP_446028.1, NP_004896.1, AQW41375.1, ADJ21808.1, CAK22382.1 XP_320690.3, AAY66580.1) of PRX6 from different species was executed to confirm the clade of LmPrx6 and to identify the motif. Evolutionary analyses for phylogenetic relationships of proteins (AEI52300.1, AAX18657.1, XP_001607910.1, AAG47823.1, AAK97814.1, XP_968419.1, ABB91779.1, ACJ53746.1, NP_446028.1, NP_004896.1, AQW41375.1, ADJ21808.1, CAK22382.1 XP_320690.3, NP_001040386.1, RZF48233.1, XP_022899820.1, XP_015373818.1, XP_014281612.1, ALC46298.1, MT563098, MT890637) were conducted in MEGA6 [38]. Prx3 of *Drosophila busckii* (ALC46298.1) was used as an outgroup.

### 2.4. Synthesis and Injection of dsLmPrx6

The dsRNA was generated by transcription using the T7 RiboMAX system (Promega, Fitchburg, WI, USA) as described in the manufacturer’s protocol in vitro. Templates for in vitro transcription reactions were prepared by PCR amplification from plasmid DNA of the cDNA clone of *LmPrx6* using the primer pairs *LmPrx6*-*2F* and *LmPrx6*-*2R* with T7 polymerase promoter sequence at the 5 ‘-end (Table 1). The length of *dsLmPrx6* was 710 bp. A total of 5 µL of *dsLmPrx6* (2 µg/µL) as the target gene, and water as a control were injected into the ventral part between the 2nd and 3rd abdominal segments of female adults within 72 h after molting under a short and long photoperiod. Details about the replicates and RNAi methods followed those of Hao et al. [7].

### 2.5. Quantitative Real-Time Polymerase Chain Reaction (qRT-PCR)

cDNA was synthesized from the RNA samples above using M-MLV reverse transcriptase and recombinant RNase inhibitor (Takara, Beijing, China). The expression levels of *LmPrx6* and the other four genes were determined by the qRT-PCR using SYBR Premix Ex Taq kit (Takara) per the manufacturer’s instructions in an ABI 7500 real-time PCR system (Applied Biosystems, Foster City, CA, USA). qRT-PCR was performed as per the following conditions: 95 °C for 10 min; 40 cycles of 95 °C for 15 s, 60 °C for 45 s. Gene expression was quantified using the 2^−∆∆Ct^ method [39], with *β-actin* as the internal control for normalization of data. The specific primers used for qRT-PCR are listed in Table 1.

### 2.6. Diapause Rate Detection

Locusts of each treatment and replication were placed in new mesh cages (25 cm × 25 cm × 35 cm) and provided with wheat grown in a greenhouse. Subsequently, 30 adult males were presented to each replicate to mate. The bottom of the cages was covered in a 5-cm layer of sieved sterile sand, with new sand given every two days. Mating occurred for about 10 days until oviposition was observed, and eggs were collected at an interval of 48 h for 10 days using a camel paint brush and transferred into paper cups (10 mm × 5 mm), where the eggs were incubated on vermiculite, before shifting to 27 °C and 60% RH to slow down the development. Around 150 eggs were obtained from 3–4 pods, which were then used in each experimental replication. Eggs were kept under 27 °C for 20 days until eclosion of the 1st instar nymphs ceased (D1). To account for non-viable eggs, all remaining uneclosed eggs were kept at 4 °C for 60 days to receive ample time to break the diapause; afterwards, they were incubated at 27 °C for 20 days and for any further 1st instar emergence (D2). The diapause rate (DR) was calculated as: DR (%) = D2/(D1 + D2) × 100%.

### 2.7. LmPTP1B, LmIR, LmAkt, and LmFOXO Phosphorylation Level Detection

Enzyme-linked immunosorbent assay (ELISA) was used to monitor and measure the quantity of insulin receptor LmPTP1B, LmP-PTP1B, LmIR, LmP-IR, LmAKT, LmP-Akt, LmFOXO, and LmP-FOXO in *L. migratoria* using the specified manufacturer’s instructions through catalogue no. SU-B97219, SU-B97220, SU-B97124, SU-B97125, SU-B97136, SU-B97137, SU-B97140, and SU-B97141 (Collodi Biotechnology Co., Ltd. Quanzhou, China). The methods were followed by Hao et al. [7]. The samples were homogenized in 1 mL of phosphate buffer saline (PBS), and the resulting suspension was subjected to ultra-sonication to further disrupt the cell membranes. After homogenates were centrifuged for 15 min at 5000 rpm, the supernatants were collected and were stored at −20 °C until being used for further analysis. All of the required reagents and samples, including micro ELISA strip plate (12 × 4 strips), standards ×6 vials (0.5 Ml × 6 vials), 3 mL of sample diluent, 5 mL of horseradish peroxidase (HRP)-conjugate reagent, 15 mL of 20 × wash solution, 3 mL of stop solution, 3 mL of chromogen solution A, 3 mL of chromogen solution B, two closure plate membranes, and a sealed bag, were prepared and properly maintained at room temperature (18 °C–25 °C) for 30 min prior to initiating the further assay procedure. We set-up standard wells, sample wells, and blank (control) wells, and then added 50 μL of standard to each standard well, 50 μL of sample to each sample well, and 50 μL of sample diluent to each blank/control well. Then, 100 μL of HRP-conjugate reagent were added to each well and covered with an adhesive strip and incubated for 60 min at 37 °C. The Micro titer plates were rinsed using Wash Buffer (1×) 4 times followed by adding gently mixed Chromogen Solution A (50 μL) and Chromogen Solution B (50 μL) to each well in succession, protected from light and incubated for 15 min at 37 °C. Finally, 50 μL of Stop Solution were added to each well. During the process, the well color changing from blue to yellow showed a proper sign and confirmation of uniformity. Colorless or green color is usually a sign of no uniformity. In such a case, the plate was then gently tapped to ensure thorough mixing. The optical density (OD) at 450 nm was read using a Micro Elisa Strip plate reader (Multiskan™ FC51119000) within 15 min of adding the Stop Solution. Standard curves of LmPTP1B, LmP-PTP1B, LmIR, LmP-IR, LmAKT, LmP-AKT, LmFOXO, and LmP-FOXO were constructed respectively and calculated accordingly to quantify the amount of sample of LmPTP1B, LmP-PTP1B, LmIR, LmP-IR, LmAKT, LmP-AKT, LmFOXO, and LmP-FOXO. The phosphorylation level of LmPTP1B, LmIR, LmAKT, and LmFOXO was then calculated as: LmP-PTP1B level =(P- PTP1B)/(PTP1B + (P- PTP1B)), LmP-IR level =(P-IR)/(IR + (P-IR)), LmP-AKT level =(P-AKT)/(AKT + (P-AKT)), LmP-FOXO level =(P-FOXO)/(FOXO+(P-FOXO)). The regression equation of the standard curve was used to determine the specificity.

### 2.8. ROS Activity Detection

Rapid ELISA-based quantification was used to detect and quantify the ROS activities in the female bodies of *L. migtatoria* using the specified manufacturer’s instructions for catalogue WLB-9124701 (Welab Biotechnology Co., Ltd., Beijing, China). The body samples were homogenized in 1 mL of PBS, and the resulting suspension was subjected to ultra-sonication (power = 20%, disrupt 3s, interval 10 s, repeat 30 times) to further disrupt the cell membranes. After this, homogenates were centrifuged for 15 min at 5000 rpm, and the supernatants were collected and stored at −20 °C until being used for further analysis. All of the required reagents and samples were prepared and were properly maintained at room temperature for 30 min prior to initiating further assay. We set-up blank (control) wells, sample wells, and standard wells. We then added 50 µL of sample diluent to each blank/control well, 50 µL of sample to each sample well, and 50 µL of standard to each standard well. Then, 50 µL of HRP-conjugate reagent were added to each well and covered with an adhesive strip and incubated for 30 min at 37 °C. The Micro titer plates were rinsed using Wash Buffer 5 times followed by adding gently mixed Chromogen Solution A (50 µL) and Chromogen Solution B (50 µL) to each well in succession, protected from light and incubated for 10 min at 37 °C. Finally, 50 µL of Stop Solution were added to each well. During the process, the well color changed immediately from blue to yellow, showing a proper sign and confirmation of uniformity. The optical density (OD) at 450 nm was read using a Micro Elisa Strip plate reader (Multiskan™ FC 51119000, Thermo Fisher Scientific Inc., Waltham, MA, USA) within 15 min of adding the Stop Solution. Standard curves of ROS were constructed and calculated accordingly to quantify the amount of ROS of each sample.

### 2.9. Catalase, Mn-SOD, Glycogen Synthase, and PEPCK Activities in the Diapause Eggs

Spectrophotometry was used to detect the catalase, Mn-SOD, glycogen synthase, and PEPCK activities in the female bodies of *L. migtatoria* using the specified manufacturer’s instructions for catalogue CAT-2-Y, SOD-2-Y, GCS-2-Y, and PEPCK-2-Y separately (Comin Biotechnology Co. Ltd., Suzhou, China). The female bodies of each treatment were homogenized in 1 mL of PBS with 0.1 g of tissue, and the resulting suspensions were subjected to ultra-sonication to further disrupt the cell (power = 20%, disrupt 3 s, interval 10 s, repeat 30 times). After this, homogenates were centrifuged for 10 min at 8000 rpm, and the supernatants were collected and were stored at −20 °C until being used for further analysis. We then added 90 µL of sample diluent to each blank/control well, 90 µL of sample to each sample well, and 90 µL standard to each standard well. In total, 240 µL of Reagent I, 6 µL of Reagent II, 180 µL of Reagent III, and 510 µL of Reagent IV were successively added to each well. In such a case, the plate was then gently tapped to ensure thorough mixing. All of the required reagents and samples were prepared and were properly maintained at room temperature for 30 min. The optical density (OD) at 240, 560, 340, and 340 nm of catalase, Mn-SOD, glycogen synthase, and PEPCK treatments were separately read using a Micro Elisa Strip plate reader (Multiskan™ FC 51119000, Thermo Fisher Scientific Inc., Waltham, MA, USA). Standard curves of catalase, Mn-SOD, glycogen synthase, and PEPCK were separately constructed and calculated accordingly to quantify the amount of those of each sample.

## 3. Results

### 3.1. LmPrx6 Cloning and dsLmPrx6 Synthesis

**Figure 2:**
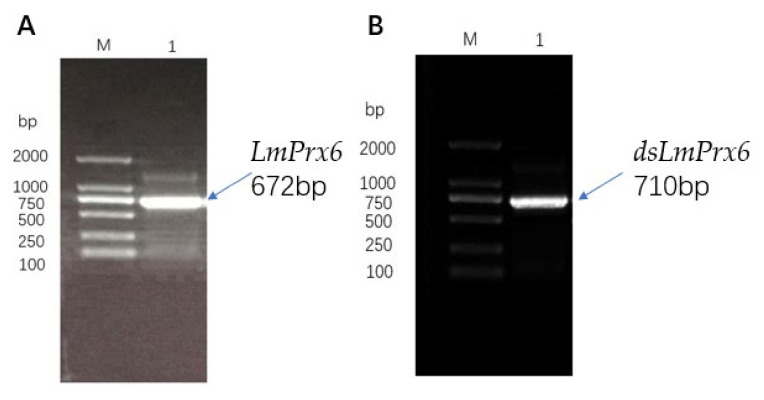
(**A**): Amplification of *LmPrx6* (672 bp). M: DL 2000 DNA Maker; 1: *LmPrx6*; (**B**): Synthesis of dsLmPrx6 (T7+LmPrx6, 710 bp). M: DL2000 DNA Maker; 1: *dsLmPrx6*.

The *LmPrx6* (GenBank accession: MT563098) was cloned from the *L. migratoria* cDNA. Results showed that the sequence of *LmPrx6* cloned was identical with the transcriptome one. The sequence of *LmPrx6* (Figure 2A) contained 672 nucleotides and the *dsLmPrx6* with double T7 strands (a total of 38 nucleotides) (Figure 2B) contained 710 nucleotides.

### 3.2. Structure and Phylogenetic Analyses of LmPrx6

**Figure 3:**
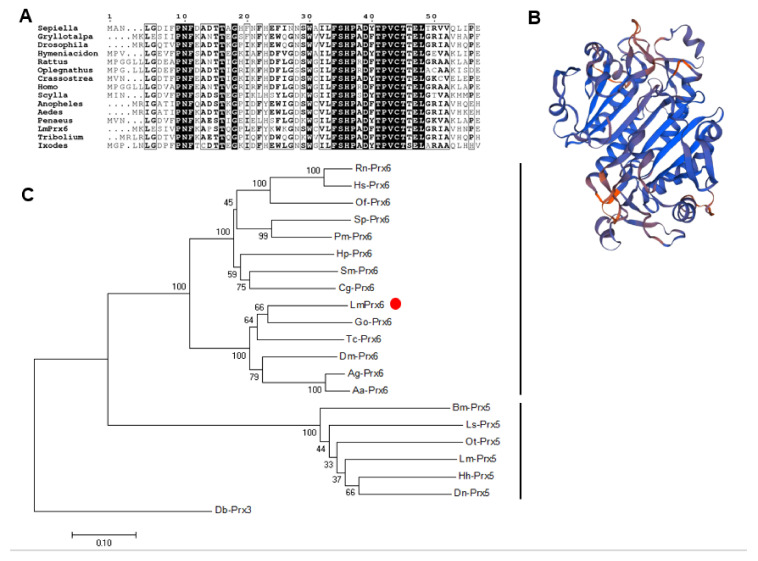
(**A**): Multiple sequence alignment with 15 sequences of PRX6 from different species; (**B**): the predicted 3-D structure of LmPrx6 with 28.18% α-helix, 19.55% extended strand, 5% β-turn, and 47.27% random coil; (**C**): phylogenetic analysis with 14 sequences of PRX6 from different species. The tree was generated with MEGA6 after CLUSTAL W alignment using the amino acid sequence of *Aedes aegypti* (AAG47823.1), *Sepiella maindroni* (AEI52300.1), *Gryllotalpa orientalis* (AAX18657.1), *Drosophila melanogaster* (AAG47823.1), *Tribolium castaneum* (XP_968419.1), *Hymeniacidon perlevis* (ABB91779.1), *Scylla paramamosain* (ACJ53746.1), *Rattus norvegicus* (NP_446028.1), *Homo sapiens* (NP_004896.1), *Penaeus monodon* (AQW41375.1), *Oplegnathus fasciatus* (ADJ21808.1), *Crassostrea gigas* (CAK22382.1), *Anopheles gambiae* (XP_320690.3), *Bombyx mori* (NP_001040386.1), *Laodelphax striatellus* (RZF48233.1), *Onthophagus Taurus* (XP_022899820.1), *Diuraphis noxia* (XP_015373818.1), *Halyomorpha halys* (XP_014281612.1), *Locust migraytoria* (MT563098), *Locust migratoria* (MT890637), and Prx3 of *Drosophila busckii* (ALC46298.1) was used as outgroup.

Phylogenetic analyses using 15 proteins demonstrated that LmPrx6 belongs to the Prx6 family and only the motif TPVCT has a Cys (Figure 3A). The secondary structure of the LmPrx6 protein was analyzed by the SOPMA online server (https://npsa-prabi.ibcp.fr/cgi-bin/npsa_automat.pl?page=/NPSA/npsa_sopma.html) and the predicted 3-D structure was analyzed by the website: https://swissmodel.expasy.org/interactive (Figure 3B). The result showed that the four secondary structures (α-helix, extended strand, β-turn, and random coil) in LmPrx6 accounted for 28.18%, 19.55%, 5%, and 47.27%, respectively. Moreover, there was no signal peptide in the protein. Evolutionary analyses were conducted in MEGA6 [38]. The evolutionary history was inferred using the neighbor-joining method [40]. The optimal tree with the sum of branch length = 3.94690523 is shown in Figure 3C. The percentage of replicate trees in which the associated taxa clustered together in the bootstrap test (1000 replicates) are shown next to the branches [41]. The evolutionary distances were computed using the Poisson correction method [42] and are in units of the number of amino acid substitutions per site. The analysis involved 21 amino acid sequences. All ambiguous positions were removed for each sequence pair. There was a total of 220 positions in the final dataset. The multiple sequence alignment is shown in Figure 3C.

### 3.3. Functional Identification of LmPrx6 by RNAi

**Figure 4:**
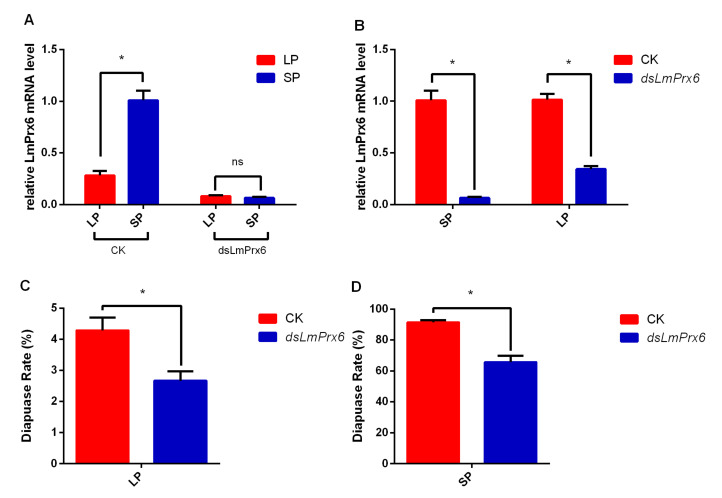
(**A**), Relative mRNA level of *LmPrx6* in the whole body of adult females under an SP (short photoperiod) and LP (long photoperiod) with and without dsLmPrx6 injection. (**B**), The comparison of the expression of LmPrx6 with and without dsLmPrx6 injection under SP and LP. (**C**), Diapause rate detected after injecting *dsLmPrx6* under LP. (**D**), Diapause rate detected after injecting *dsLmPrx6* under SP. All results were expressed as means ± standard error (SE) of the three replicates. * Indicates probability level of *p* < 0.05 by Student’s *t*-test.

A previous study [22] on the transcriptomes of adults under long (16:8 L:D) and short (10:14 L:D) photoperiods showed that *LmPrx6* was involved in diapause regulation in *L. migratoria*. To verify the function of *LmPrx6* on locust diapause, the primary relative mRNA level of *LmPrx6* was determined in *L. migratoria* treated under both short photoperiods (SPs) and long photoperiods (LPs). Results showed that *LmPrx6* expression of locusts treated by SP was 3.5 times higher than that of LP (Figure 4A). *dsLmPrx6* was then injected into female *L. migratoria* adults to knock down *LmPrx6* under LP and SP to identify the *LmPrx6* function, followed by confirmation of RNAi efficiency via qRT-PCR. There is no significance in locusts under LP and SP when LmPrx6 was knocked out (Figure 4A). The variable expression of *LmPrx6* with significant (*p* < 0.05) change in *dsLmPrx6* treatments and CK (ddH_2_O) under both photoperiods indicated the acceptability of RNAi efficiency of *LmPrx6* (Figure 4B). Under LP, the average egg diapause rate (2.6%) in the *dsLmPrx6* treatment was significantly lower (Figure 4C) than the CK (4.2%). Similarly, under SP, the average egg diapause rate (65.6%) in the *dsLmPrx6* treatment was significantly lower (Figure 4D) than that of the control (91.4%). This shows that the knockdown of *LmPrx6* could inhibit diapause of *L. migratoria* under both photoperiods.

### 3.4. Impact of LmPrx6 on the Phosphorylation Level of Downstream PTP1B, IR, AKT, and FOXO

A prior study in our lab showed that ISP plays a vital role in regulating locust diapause [43]. The phosphorylation levels of proteins involved in ISP, including LmIR, LmAKT, LmFOXO, and LmPTP1B, were analyzed. ELISA was performed to determine the phosphorylation level of these four key proteins in the ISP.

**Figure 5:**
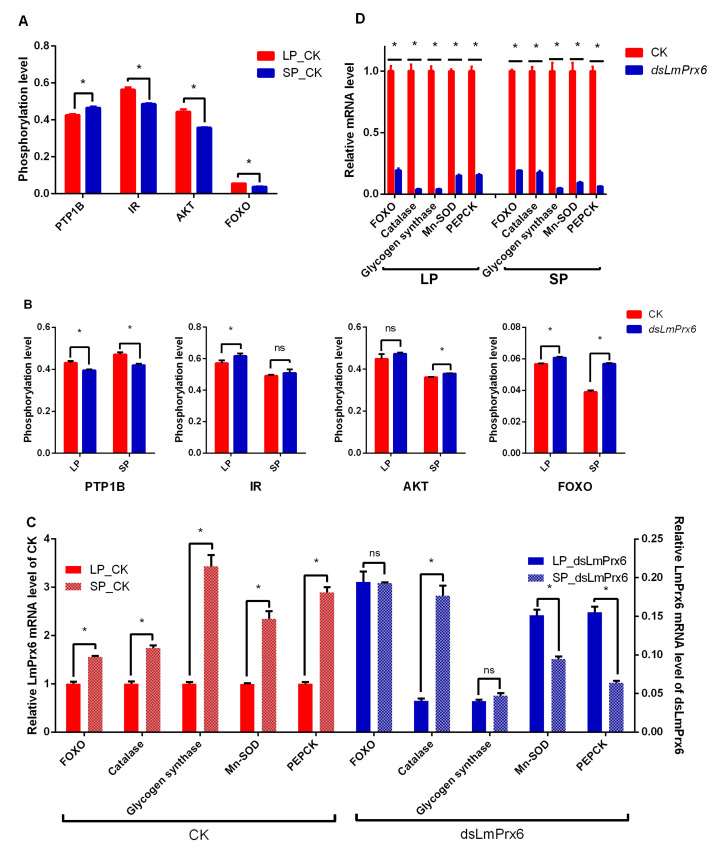
(**A**), Phosphorylation level of LmPTP1B, LmIR, LmAKT, and LmFOXO under both long and short photoperiods (LP, SP). (**B**), Phosphorylation level of LmPTP1B, LmIR, LmAKT, and LmFOXO in the control compared to *dsLmPrx6* treatment under both LP and SP. All results were expressed as means ± standard error (SE) of the three replicates. (**C**), Relative mRNA level with and without dsLmPrx6 injection of FOXO, catalase, glycogen synthase, Mn-SOD, and PEPCK under LP compared to SP. (**D**), Relative mRNA level of FOXO, catalase, glycogen synthase, Mn-SOD, and PEPCK in CK compared to *dsLmPrx6* treatment under both LP and SP. * Indicates probability level of *p* < 0.05 by Student’s *t*-test.

Results showed that LmPTP1B (t = 5.18766, *p* = 0.0065716) was phosphorylated at a higher level in ISP while LmIR (t = 6.9158, *p* = 0.00229376), LmAKT (t = 6.49266, *p* = 0.00290215), and LmFOXO (t = 29.9989, *p* < 0.0001) were phosphorylated at a lower level in *L. migratoria* under SP than that of LP (Figure 5A). In addition to LmPTP1B, the phosphorylation of LmIR, LmAKT, and LmFOXO in ISP was inhibited in locusts under SP; however, only the phosphorylation level of LmIR (t = 1.42263, *p* = 0.227919) was non-significant in the *dsLmPrx6* treatment compared to CK (ddH_2_O), whereas LmPTP1B (t = 6.94619, *p* = 0.00225642), LmAKT (t = 7.23101, *p* = 0.00194051), and LmFOXO (t = 22.2478, *p* < 0.0001) were significantly higher. In contrast, the phosphorylation level of LmPTP1B (t = 6.45263, *p* = 0.00296948), LmIR (t = 3.22689, *p* = 0.0320699), and LmFOXO (t = 7.35111, *p* = 0.00182379) were significantly higher in the *dsLmPrx6* treatment compared to the control under LP, whereas the LmAKT (t = 1.69679, *p* = 0.164975) expression was non-significant (Figure 5B). Results suggested that LmPrx6 probably influences the phosphorylation of PTP1B and FOXO under both SP and LP.

### 3.5. Impact of LmPrx6 on the mRNA Level of Genes Downstream of Foxo

Previous studies showed that stress tolerance [44] and metabolic pathways [45] were regulated by FOXO signaling. The mRNA levels of downstream genes, catalase, and Mn-SOD in stress tolerance and glycogen synthase and PEPCK in the metabolic pathway were detected by qRT-PCR. The results (Figure 5C) showed that the mRNA levels of FOXO (t = 12.4162, *p* = 0.000241907), catalase (t = 9.5844, *p* = 0.000662234), glycogen synthase (t = 10.1971, *p* = 0.000521083), Mn-SOD (t = 8.21129, *p* = 0.00119878), and PEPCK (t = 16.5777, *p* < 0.0001) under LP were all higher than under SP. When LmPrx6 was knocked down, there is no significance of FOXO (t = 0.13197, *p* = 0.90138) and glycogen synthase (t = 1.73395, *p* = 0.157951) under LP and SP whereas the expression level of Mn-SOD (t = 7.26409, *p* = 0.00190746) and PEPCK (t = 12.0495, *p* = 0.000272016) were significantly higher under LP versus SP (Figure 5C). The expression level of catalase under LP was significantly lower than that under SP (t = 10.2502, *p* = 0.000510681) (Figure 5C). Moreover, the expression levels (Figure 5D) of FOXO (t = 18.7081, *p* < 0.0001), catalase (t = 18.7171, *p* < 0.0001), glycogen synthase (t = 26.1373, *p*< 0.0001), Mn-SOD (t = 51.1554 *p* < 0.0001), and PEPCK (t = 22.6859, *p* < 0.0001) were significantly lower in the *dsLmPrx6* treatment compared to the CK (ddH_2_O) under SP, whereas FOXO (t = 67.9437, *p* < 0.0001), catalase (t = 23.1976, *p* < 0.0001), glycogen synthase (t = 13.8645, *p* = 0.000156898), Mn-SOD (t = 13.0012, *p* = 0.000201965), and PEPCK (t = 24.9971, *p* < 0.0001) were also significantly lower in the *dsLmPrx6* treatment compared to the CK (ddH_2_O) under LP. These findings indicated that knocking down *LmPrx6* could inhibit the transcription of FOXO, catalase, glycogen synthase, Mn-SOD, and PEPCK.

### 3.6. ROS Activity Regulated by LmPrx6

**Figure 6:**
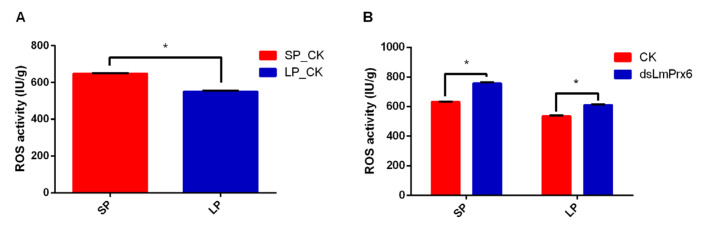
(**A**), ROS activity in the whole body of adult females under long and short photoperiods. (**B**), ROS activity detected in the whole body of adult females after injecting *dsLmPrx6* under SP and LP. All results were expressed as means ± standard error (SE) of the three replicates.* Indicates probability level of *p* < 0.05 by Student’s *t*-test.

To identify the effect of *dsLmPrx6* injection on ROS, *dsLmPrx6*-treated and controlled locusts under both SP and LP were determined subsequently. Results showed that the ROS activity of locusts treated by SP was significantly (t = 20.2633, *p* < 0.0001) higher than that under LP-treated locusts (Figure 6A). Under SP, the ROS activity was 632.222 IU/g in the *LmPrx6* treatment, significantly (t = 18.3069, *p* < 0.0001) lower (Figure 6B) than that of CK (ddH_2_O) (757.096 IU/g). In a similar manner, the ROS activity in the *LmPrx6* treatment (535.007 IU/g) was also significantly (t = 14.6086, *p* = 0.000127722) lower than that of the control (611.516 IU/g) under LP (Figure 6B). These results above indicate that LmPrx6 probably positively regulated the ROS activity and subsequently promoted diapause induction under LP and SP.

### 3.7. Catalase, Mn-SOD, Glycogen synthase, and PEPCK activities Regulated by LmPrx6

**Figure 7:**
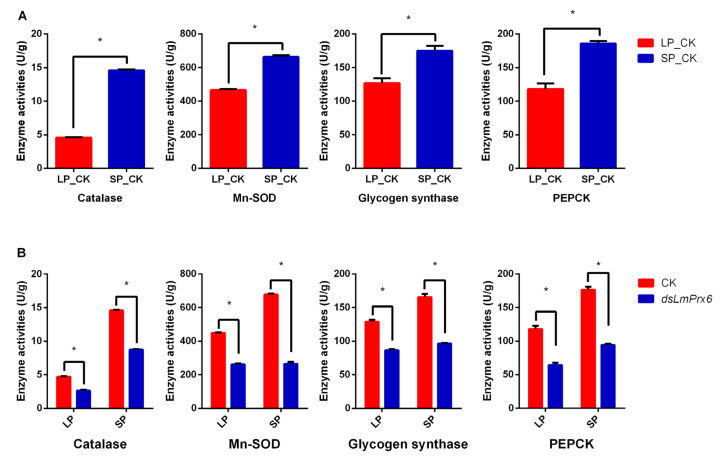
(**A**), Catalase, Mn-SOD, glycogen synthase, and PEPCK activities in the whole body of adult females under long and short photoperiods. (**B**), The comparison of catalase, Mn-SOD, glycogen synthase, and PEPCK activities detected in the whole body of adult females with and without injection of *dsLmPrx6* under SP and LP. All results were expressed as means ± standard error (SE) of three replicates. * Indicates probability level of *p* < 0.05 by Student’s *t*-test.

To find more evidence, we measured the activities of four diapause-related enzymes (catalase, Mn-SOD, glycogen synthase, and PEPCK) downstream of LmFOXO. Results showed that catalase (t = 59.9965, *p* < 0.0001), Mn-SOD (t = 16.1754, *p* < 0.0001), glycogen synthase (t = 4.59764, *p* = 0.0100488), and PEPCK (t = 7.4334, *p* = 0.00174879) demonstrated higher enzyme activities under SP than that of under LP (Figure 7A). Furthermore, when we knocked down LmPrx6, catalase (t= 11.6572, *p* = 0.000309577), Mn-SOD (t = 26.5529, *p* < 0.0001), glycogen synthase (t = 13.2792, *p* = 0.000185874), and PEPCK (t = 8.93517, *p* = 0.000867595) under LP showed decreased enzyme activities with the same consequences of catalase (t = 76.2434, *p* < 0.0001), Mn-SOD (t = 31.6613, *p* < 0.0001), glycogen synthase (t = 15.2925, *p* = 0.000106648), and PEPCK (t = 17.6573, *p* < 0.0001) under SP (Figure 7B).

## 4. Discussion

Insects enter a static stage by sensing changes in the external environment, such as the photoperiod, temperature, and food volume [2], often accompanied by inhibition of metabolism, for example, slow growth or even stagnation, decreased respiratory rate, etc. [3]. *Locusta migratoria* exhibits facultative egg diapause, and egg diapause has been shown to be influenced by the maternal photoperiod in *L. migratoria*. Although the diapause varied in different geographic locations in a previous study [1], our Huanghua strain (collected in Huanghua, Tianjin, China (38°49′N, 117°18′E)) enters diapause under short photoperiod (SP) conditions. Before the current experiment, transcriptome analysis of the diapause and non-diapause phenotypes showed that *LmPrx6* was involved in the diapause maternal locusts, but the mechanism was not clear.

LmPrx6 was verified by the alignment with Prx6 proteins from 14 other species and the phylogenetic tree showed that the Prx6 family members are highly conserved. All 15 proteins have the same TPVCT motif, indicating that the only conserved Cys is probably the active one.

Previous studies showed that the presence of Prx6 inhibits the activity of ROS in vivo [25,46], while excess ROS can directly inactivate the transcription factor FOXO downstream of the ISP by phosphorylation [47], causing weaker signaling and the lower diapause rate. In the current study, qRT-PCR was initially applied to check the mRNA level of *LmPrx6*. The *LmPrx6* expression under SP was significantly higher than under the long photoperiod (LP) (Figure 4A). Thus, these results suggest that *LmPrx6* might positively regulate diapause. To test this hypothesis, RNAi was performed to check *LmPrx6* functions. One previous researcher showed that RNAi sensitivity in migratory locust varies because of strains rather than because of different genes in one species [48]. Our results showed that *LmPrx6* expression decreased by 93.6% and 66.1%, respectively, under SP and LP after knocking down *LmPrx6* (Figure 4B). This suggests that the RNAi efficiency is acceptable in the Huanghua strain. Moreover, the diapause rate significantly decreased after *dsLmPrx6* injection (Figure 4C). The RNAi result was identical to the qRT-PCR outcome. It revealed that *LmPrx6* promotes diapause induction in *L. migratoria* under both photoperiods.

To confirm the relationship between LmPrx6 and the ISP at the protein level, the phosphorylation level of LmFOXO was determined in CK (ddH_2_O) and *dsLmPrx6*-treated locusts under both LP and SP. Results demonstrated that the phosphorylation level of LmFOXO, which represents LmFOXO inactivity, increased after knocking down *LmPrx6*. Results also indicated that *LmPrx6* could activate LmFOXO to induce diapause of *L. migratoria*. Moreover, we detected the upstream protein LmPTP1B of the ISP [49] and found that the phosphorylation of LmPTP1B protein was significantly reduced, while LmFOXO phosphorylation was significantly increased. The phosphorylated LmFOXO, moving from the nucleus to the cytoplasm, cannot regulate the expression of fat synthesis genes and the ISP was inhibited. In contrast, when LmPrx6 was attenuated, the mRNA level of FOXO decreased sharply (Figure 5D), indicating that LmPrx6 probably functions at the transcriptional level.

In contrast, a large amount of ROS can inhibit the activity of PTP1B [50], which plays an essential role in balancing IR and insulin receptor substrate (IRS) [16,17], and ultimately increases FOXO activity [51]. A previous report also showed that the ROS level increased when diapause induction of *L. migratoria* occurred under SP [37]; thus, our recent findings are consistent with our previously verified results for *rai1*. Additionally, we found that ROS activities were increased after knocking down *LmPrx6* under both LP and SP. This expression was almost similar to the knockdown of *rai1* under SP [22]. The result indicated that *LmPrx6* inhibited LmFOXO activity, which ultimately induced locust diapause.

Enhanced stress tolerance is one of the important features of diapause, and is essential for successful overwintering. Several lines of evidence suggest that genes encoding two antioxidant enzymes, catalase and superoxide dismutase-2, are critical in generating these characteristics during diapause in overwintering adults of the mosquito *Culex pipiens* [45]. Mn-SOD has already been identified as an important target downstream gene of FOXO in mice [52] and nematodes [53]. In *L. migratoria*, the expression of Mn-SOD and SOD was significantly higher in samples under SP than under LP [54]. The mRNA expression levels of catalase and Mn-SOD in the whole body of *L. migratoria* and the enzyme activities of these two proteins were consistent in tendency (Figure 5C and Figure 7A). After knocking down *LmPrx6,* the mRNA level of these genes and enzyme activities of these proteins were both decreased (Figure 5D and Figure 7B). It suggested that LmFOXO could positively regulate catalase and Mn-SOD, which may be delivered to eggs, hence enhancing the stress tolerance of diapause eggs. It also suggests that catalase and Mn-SOD are probably critical links between the ISP and adult diapause induction

Efficient storage of energy reserves during diapause is crucial not only for surviving prolonged periods of developmental arrest but also for maximizing reproductive success when diapause has terminated and development resumes [45]. Glycogen synthesis is regulated by glycogen synthase kinase-3 (GSK-3) in response to insulin, which was involved in diapause processing in *Bombyx mori* eggs [55]. Insulin stimulation results in the phosphorylation and inactivation of GSK-3, rendering it incapable of inhibiting glycogen synthase activity, thus leading to increased glycogen synthesis [55,56,57,58]. In *Aphidius gifuensis*, glycogen synthase involved in trehalose synthesis was differentially expressed in diapause and non-diapause *A. gifuensis* [59]. In this paper, the mRNA expression levels and the enzyme activity of glycogen synthase (Figure 5D and Figure 7B) of female adults were both significantly downregulated with the attenuation expression of LmFOXO after *LmPrx6* interference. These results suggested that ds*LmPrx6* might indirectly inhibit the delivery of sufficient nutrition to eggs to complete the diapause process. Phosphoenolpyruvate carboxykinase (PEPCK) is part of the gluconeogenesis pathway, and has higher expression levels during diapause in *Sarcophaga crassipalpis* [60]. Low expression of PEPCK is related to levels in pyruvate biosynthesis, resulting in low pyruvate diapause-destined pupae to induce lifespan extension or diapause via low metabolic activity [61]. In our results, the mRNA expression levels and the enzyme activity of PEPCK (Figure 5D and Figure 7B), which might contribute to energy storage, were also significantly downregulated with the reduction of LmFOXO after *LmPrx6* interference.

## 5. Conclusions

We successfully cloned *LmPrx6* for the first time from *L. migratoria*. Structure and phylogenetic analyses showed that LmPrx6 was highly conserved in different species, with the motif TPVCT probably containing the only active Cys. Results showed that the expression of *LmPrx6* was higher in female locusts under diapause conditions than non-diapause females. This consequence is consistent with transcriptome data. The female adults sensing the short photoperiod cause the increase of LmPrx6, which inhibits proteins in ISP through ROS. Finally, the activities of diapause-related enzymes (catalase, Mn-SOD, glycogen synthase, and PEPCK) were also significantly enhanced by LmPrx6. The regulatory mechanism of LmPrx6 promoting diapause in *L. migratoria* is associated with the decreased activities of ISP proteins and enhanced activities of FOXO and diapause-related proteins. When autumn locusts lay eggs, the transgenic approach and utilization of *dsLmPrx6* could be used as a green pesticide to control locusts. The targeting of LmPrx6 in this manner could provide an efficient and environmentally sustainable approach to reduce the agricultural damage caused by *L. migratoria*.

## Figures and Tables

**Table 1:** List of specific primers used and synthesized for the current study.

Primers	Primer Sequences (5′–3′)	Purpose
*LmPrx6-1F* *LmPrx6-1R*	ACGTAGTTGTTGCCGGAG	Clone of the target gene
	CGCTACAGAGAGAAGTCTACA	
*LmPrx6-2F* *LmPrx6-2R*	TAATACGACTCACTATAGGACGTAGTTGTTGCCGGAG	Synthesis of the dsRNA
	TAATACGACTCACTATAGGCGCTACAGAGAGAAGTCTACA	
*LmPrx6-3F*	TGGAAAGGAAACTCGTGG	RT-qPCR
*LmPrx6-3R*	TTGTCACAGGAGAGAGCCA	
*foxo-F*	AGAACTCGATCCGGCACAAC	
*foxo-R*	CGCCTCCACCTTCTTCTTGA	
*Catalase*-F	GGTATTTGGGATTTGGTGG	
*Catalase*-R	GGGTTGTCTCTGGTCTAAGTG	
*Glycogen synthase*-F	AAAGTTCCTCGCTCTCCACG	
*Glycogen synthase*-R	ACATCAGCACCCTTGTTTCC	
*Mn-SOD*-F	CAGACCAACGCTACTCTCGC	
*Mn-SOD*-R	TAATGACCTCCCAAATGGCG	
*Phosphoenolpyruvate*-F	ATTTAGAAACGGGAGGCAAG	
*Phosphoenolpyruvate*-R	AGGTGGATTACTCGGATGAC	
βactin-F	GTTACAAACTGGGACGACAT	qPCR reference gene
βactin-R	AGAAAGCACAGCCTGAATAG	

## Abbreviations

ISP	Insulin Signaling Pathway
Prxs	Peroxiredoxins
ROS	Reactive Oxygen Species
FOXO	forkhead box protein O
PTP1B	Protein-Tyrosine Phosphorylase 1B
AKT	RAC serine/threonine-protein kinase
IR	insulin receptor
Mn-SOD	Mn superoxide dismutase
PEPCK	phosphoenolpyruvate carboxy kinase
SP	Short photoperiod
LP	Long photoperiod
DR	Diapause rate
ELISA	Enzyme-Linked ImmunoSorbent Assay
RNAi	RNA interference
